# The London Is Bold

**Published:** 1890-09-20

**Authors:** 


### The Hospital

A Weekly Journal of

Science, Medicine, Nursing, and Philanthropy.

*For Hospitals, Asylums, Medical Practitioners, Students, Nurses, Families, Parishes, Congregations, and the Charitable Public.*

Vol. VIII.—No. 208.] [September 20, 1890.

### The London is Bold.

The London Hospital has chosen the present moment, when certain departments of its management are the subjects of public comment and criticism, to make an appeal to the charitable to raise its annual subscription income to £34,000. The London is bold and it is wise. It has been affirmed on the one hand that there has been a dead set made against the hospital by an organised opposition; and the affirmation has been stoutly denied on the other. But whatever be the truth of the matter, this much at least is patent to all men; that certain persons have been very willing to bring charges against the hospital, and that they have made their charges as full and complete as possible. What then do the charges amount to when they are all made? Are they small or are they great? Are they important or are they trifling? Compared with the magnitude and importance of the whole work done at the London Hospital, they are so small as to be utterly insignificant. They do not affect the honesty of the management; they do not affect its economy; they do not affect its efficiency. There is no charge of misuse of charitable funds, nor is there any hint of unkindness to patients or of want of competence in medical or surgical treatment. The whole accusation, not the proved statement, but the accusation, amounts to no more than this, that at some not very recent period in the history of the hospital, certain nurses were overworked, and they were not delicately fed. If the charge were true to the very minutest detail, it is but a small matter which could be remedied without trouble, and for which no one need be severely blamed. But it is not proved to be even true. On the contrary, the weight of responsible evidence leads to the entirely opposite conclusion, that the nurses of the London hospital are as liberally fed, and as reasonably worked as ever nurses may expect to be at an institution of the kind.

Enough has been said by way of explanation. We turn now to the appeal for largely increased annual subscriptions. It is not necessary to give any account of the work of the London Hospital to those who know the institution, and who are already its active friends. They are so convinced of the rare value of its work, and of the stern, pitiful, and indescribable necessities of the neighbourhood in which it is placed, that every one of them who has money to spare will hasten to give it with constantly expanding liberality. But hospitals, like churches and businesses, must make new friends as well as retain the old; and our object in this article is not to preach to the converted, but to ask those who have hitherto had no connection with the London Hospital to give a few minutes' intelligent and sympathetic consideration to the work it is doing and to the people who seek its aid. Some of the great facts that strike a person experienced in hospital affairs when he thinks of the London are these: The hospital is the largest in the kingdom, and it is placed in the midst of the largest and poorest population. It contains eight hundred beds, which are almost always full and sometimes require to be added to; and it treats more than a hundred thousand different persons every year; whilst its students attend between two and three thousand cases of confinement at their own homes annually! Now, what does this mean to the struggling, half-fed, ill-paid, and stifled men, women, and children who live in the slums of the East-end of London? We cannot describe what it means; and if we did attempt anything like an adequate description, the reader would think we were trying to pile on masses of mere unhealthy sentimentality. The other day, in Tunbridge Wells, a poor woman, who was the sole support of her family of five children, was taken suddenly ill, and sent for the club doctor. He did not go; upon which she sent for the parish doctor, and he refused to attend. In a few hours the poor woman was dead, and her five motherless children were left to the tender mercies of a world that is often very cold and unfeeling. If it were not for the London Hospital, with its eight hundred beds and its large out-patient department, how many cases like this poor woman's might happen at the East-end of London every day? Sometimes ten, sometimes fifty, sometimes a hundred or more. All Tunbridge Wells was moved to indignation by the single case we have mentioned; and not only Tunbridge Wells, but newspaper writers in every part of the country. The plain truth is that neither gentle-hearted subscribers, nor nurses, nor doctors, nor even the poor sufferers themselves of the East of London realise in any but the faintest degree the hope, the comfort, the help, the safety, and the millionfold blessing of the great hospital which is in their midst. When we think of it; of its wonderful work; of its noble physicians and surgeons, for some of them are and have been truly noble; of its practical, experienced, shrewd, kindly, and perhaps incomparable, house governor, who has spent nearly forty years of his life in conscientious and devoted service to the hospital; when we think, too, of the General Committee, and still more, of the House Committee, who give up so much of their time and of their best capacities to the welfare of the institution, we are astonished and indignant that even such irresponsible persons as a handful of girl nurses should have dared to run the risk of injuring so noble an institution on such trivial grounds; and, still more, that they should have found even so many as a dozen supporters among older and more experienced persons.

Happily the English people are adepts in the arts of common sense. They are quite able to appreciate clever speeches and fine spun arguments. But they are positively deaf and blind to any sort of consideration whatsoever which sets itself in opposition to a plain and palpable fact. The London Hospital, its history and its work, forms one of the noblest of the many noble pages in the story of English philanthropy. Circumstances have made it a noble story. The hospital was not built as some are for the convenience and glorification of medical men. It was built for the distressed poor, in the midst of the poor, and far away from the regions of luxury and ease. Its physicians and surgeons cannot be mere dilettante occasional visitors; they are obliged to be men in earnest, and have always been compelled to grapple daily with the sternest facts of life and death. The members of the committee and the official managers have never been in the position of having an overflowing exchequer and half their beds empty. On the contrary, suffering, pain, poverty, and misery of all kinds have crowded round their doors, whilst resources have been slow to increase, and have never been in the least commensurate with the overwhelming magnitude of the work. All these things have conspired to make the history of the London Hospital one long, steadfast, and heroic struggle against disease and death in their most terrible and appalling forms. Faults! undoubtedly there may have been faults now and again in the details of such a life story. But what and where are the deeds of evil which any man or woman would care to single out in comparison with the 150 years' work of this noble and beautiful medical charity? To a noble people we confidently appeal on behalf of one of the noblest of its own institutions. It is one of the worthiest of the many worthy children of this great nation; of this heroic people whom Milton was proud to extol; and one which sheds the most brilliant and amiable lustre on its parentage. The philanthropic mother would defend her child if it needed defence. But the stalwart robustness of the London Hospital asks for no help of that poor kind. It only says: "See how great is the work I have to do; see my willingness to do it; come and help me."

